# Ancillary Procedures to Facelift Surgery: What has Changed?

**DOI:** 10.1093/asjof/ojad063

**Published:** 2023-08-16

**Authors:** Michael J Stein, Sherrell J Aston

## Abstract

The facelift remains the gold standard for rejuvenating the aging face. Many surgical techniques exist in the surgeon's armamentarium that vary based on scar burden, extent of skin undermining, and manipulation of the superficial musculoaponeurotic system. Yet, existing strategies predominantly address the mobile face and have limited effect on fixed zones such as perioral, periorbita, and forehead. Multiple ancillary techniques have therefore been developed to address this therapeutic gap in facial rejuvenation. The most popular techniques today include dermabrasion, lasers, chemical peels, and radiofrequency devices. All have demonstrable safety and efficacy and are chosen based on the patient's unique anatomical presentation, comfort level, and tolerability of recovery time. Surgeons are ideally equipped with the tools and skills to offer all modalities and then tailor their treatment to the specific patient's anatomy. Herein, we review the most effective ancillary procedures of the facelift and describe an evolution of their use in our practice.

**Level of Evidence: 5:**

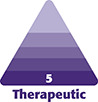

An evolving knowledge of the anatomical changes that occur with facial aging has enhanced aesthetic outcomes in facelift surgery. Adjunct surgical procedures now play a central role in the modern facelift. A well-defined neck and jawline are enhanced with platysmal tightening and debulking of fat, muscle, and gland. Facial fat grafting restores deflation and descent of the midface to restore an inverted triangular appearance. Blepharoplasties and brow lift surgery restore a youthful brow and lid shape. Lip lifts correct upper lip lengthening and rolling in of the vermilion. Minimally invasive skin resurfacing devices remove epidermal dermatoses and regenerate collagen to improve skin quality. Neuromodulators paralyze muscles to minimize movement and reduce undesired skin creases.

Facial aging results from a complex and multifactorial interplay between environment, genetics, and metabolic changes. These changes are best contextualized by appreciating the layered anatomy of the face. An understanding of the predictable morphologic changes to this layered anatomy is necessary to choose the appropriate ancillary facelift procedure.

## ANATOMICAL LAYERS

### Skin

The first layer is the skin, composed of the epidermis (a continuously differentiating layer of keratinocytes with interspersed pigment-producing melanocytes) and dermis (a collagen-rich extracellular matrix secreted by fibroblasts with a rich vascular plexus). Extrinsic insults (ultraviolet radiation, smoking, and pollution)^1,2^ generate reactive oxygen species, which lead to cellular damage and gradual thinning of these layers.^3-5^ As the layers thin, they lose their capacity to maintain static stability during muscular contraction leading to wrinkles.

In the aged epidermis, slower cell turnover leads to shorter and wider differentiating keratinocytes and a thicker and more dehydrated stratum corneal layer.^6-8^ Dry, scaly skin prone to cracks, and fissures results in an aged appearance. Tyrosinase-mediated melanogenesis in the epidermis also contributes to hyperpigmentation and melasmas that are common to the aging face.^9^ At the epidermal–dermal junction, there is loss of dermal papillae,^10^ leading to flattening, loss of resistance to shearing forces,^11^ and increased risk for epidermal–dermal separation, all of which produce wrinkles.^12^ The thickness of the dermal layer also thins, becoming more mobile and susceptible to damage. A declining number of fibroblasts and decreased vascularity^6,13^ lead to decreased collagen and elastin synthesis and a more disorganized arrangement of collagen bundles.^14,15^

To varying degrees, skin resurfacing modalities such as lasers, chemical peels, and dermabrasion function to either remove and regenerate the epidermis or promote collagen regeneration in the dermis. Antiaging skin creams, on the contrary, are predicated on maintaining the stability of the epidermis and dermis—in the epidermis, by maintaining its normal barrier and immune function and reducing transepidermal water loss, and in the dermis, by maintaining collagen integrity, promoting collagen regeneration, and maintaining (and providing) substrates for extracellular matrix maintenance.

### Subcutaneous Fat

The second layer is the subcutaneous tissue, composed of the superficial and deep fat compartments^16^ of adipocytes, providing volume and structural support to the skin. The fat is surrounded by a fibroseptal network (FSN; also known as retinacula cutis) which bridges the underlying superficial musculoaponeurotic system (SMAS) to the dermis. The thickness of the adipose layer and the density and integrity of the FSN vary topographically across the face. The upper lip, for instance, has a relative paucity of subcutaneous tissue and short, dense FSN fibers leading to less gravity-related ptosis, compared to the malar eminence, with thick fat compartments and longer FSN fibers that are more prone to attenuation with aging. Descent and deflation of the facial fat compartments are hallmarks of the aging face and usually occurs at the nasolabial fold, labiomental crease, and jowl.^17-19^

The treatment of the subcutaneous layer has been primarily surgical. FSN attenuation is addressed by dividing the FSN fibers above the SMAS during facelift flap elevation, then mobilizing and securing it proximally to promote subcutaneous scarring and adherence. It can also be addressed nonsurgical, with radiofrequency devices that contract the FSN.^20^ Fat atrophy is additionally addressed by facial fat grafting to augment deflated and descended fat compartments.^21^

### SMAS and Mimetic Muscles of the Face

The third layer of the face is the SMAS, a fascial layer of connective tissue that covers the mimetic muscles of the face. Age-related degradation of collagen and elastin leads to its increased compliance and ptosis with time. Muscles of facial expression insert into the dermis so repeated contraction result in static rhytids of the face and a hyperdynamic facial expressions.

Treatment of the SMAS is primarily addressed with facelift surgery. A variety of techniques for SMAS tightening have been described, including plication, SMASectomy, SMAS flaps, and mobilization of skin and fat as a composite.

### Sub-SMAS Space

The fourth layer is the sub-SMAS space, which is a series of soft-tissue spaces bordered by the retaining ligaments of the face at which the facial nerve branches typically traverse from deep to superficial to innervate the mimetic muscles of the face (all along their deep layer except mentalis, levator anguli oris, and buccinator). Adherence zones in Layer 4 are key suture fixation points during facelift. The so-called Deep Plane is commonly entered in facelift surgery in order to mobilize the SMAS and improve the final cheek and neck contour. Anatomically, any technique that undermines the SMAS is in the deep plane.

### Deep Fascia, Periosteum, Bone

The final layer of the face is the deep fascia, which centrally is comprised of the periosteum overlying bone, and laterally becomes the superficial muscular fascia of muscles of mastication (temporalis and master). Facial bones resorb in a predictable fashion with age. Bone creates the supportive platform for the overlying soft tissue; hence, its resorption is thought to contribute to the descent of the fat pads and muscle.^22^ Orbital bone recession deepens the lid–cheek junction/tear trophs and lead to senile enophthalmos.

While custom implants can be used in areas of bony resorption, concomitant eyelid, brow lift, and facial fat grafting surgery remain the mainstay to counteract facial changes from boney resorption. Subperiosteal facelifts have been described yet remain uncommon today.

## FACELIFT SHORTCOMINGS

The forehead, lids, and perioral region are minimally improved during facelift surgery. The forehead develops static transverse rhytids from the frontalis, vertical glabellar rhytids from the corrugators, and transverse glabellar rhytids from the procerus. Increased skin laxity leads to brow ptosis, upper and lower lid excess, and “crows feet” wrinkles from repeated contraction of the orbicularis oculi. Skin laxity, dermal thinning, and FSN attenuation of the upper lip lead to lengthening of the prolabium, while repeated orbicularis oris contraction results in perioral rhytids (so-called smokers’ lines).

While a variety of concomitant surgical procedures such as blepharoplasty, brow lifts, and lip lifts can help address such problems, nonsurgical ancillary procedures have become commonplace during facelift surgery to resurface the skin and improve skin quality and appearance. Ideally, plastic surgeons are equipped with the equipment and techniques to choose the appropriate modality and tailor their approach to the specific patient's anatomy, desires, and tolerability of recovery.

Herein, we review an evolution of the most common facelift ancillary procedures used in the last 47 years of the senior author's practice—all of which have been used as a stand-alone facial rejuvenation technique as well.

## FACELIFT ANCILLARY PROCEDURES

### Dermabrasion

This hand-held rotary device can be controlled by the surgeon to mechanically debride the epidermis and papillary dermis, creating a controlled depth injury that promotes reepithelization and collagen remodeling. The epidermis is avascular, so the appearance of pinpoint bleeding indicates the entrance into the papillary dermis and more confluent bleeding entrance into the reticular dermis. The target depth is the superficial or midreticular dermis. When performed at the appropriate depth, the rich vascular and adnexal network stimulates an inflammatory response, promoting tissue remodeling in such a way that new skin is smoother and firmer, with moderate rhytids effaced.

Benefits of dermabrasion include its low, fixed cost of operation. Disadvantages include surgeon-dependent results, a relatively ambiguous and imprecise depth of penetration, prolonged recovery (up to 4 weeks of significant scabbing and up to 8 weeks of erythema) and its higher risk profile, including infection, permanent pigmentary changes, and skin necrosis. Operator risks exist as well, as the rotating device aerosolizes blood which can be inhaled by the surgeon.^23^ The ideal candidate for dermabrasion has moderate/deep perioral rhytids, Fitzpatrick I and II skin types, has not undergone ablative facial resurfacing procedures in the past, and agrees to tolerate a prolonged healing time away from the public eye.

### Ablative Lasers

Ablative lasers such as carbon dioxide (CO2) and erbium yttrium aluminum garnet (ER:YAG) provide energy in the form of photons that are absorbed by water in the skin and then emit thermal energy, destroying the surrounding tissue and creating an inflammatory response that promotes regeneration. The benefits of lasers are increased precision and reproducibility of application depth by setting a specific pulse duration, energy, and wavelength on the machine. Disadvantages include the high fixed and maintenance costs to the provider, a prolonged recovery for the patient (with higher erythema score than dermabrasion at 1 month^24^) and the risk of permanent pigmentary changes, infection, skin necrosis, milia, and even blindness.^25-29^ Like dermabrasion, the ideal candidate for laser abrasion has moderate perioral rhytids, Fitzpatrick I and II skin types, has not undergone facial resurfacing procedures in the past, and will tolerate a prolonged healing time.

### Chemical Peels

Chemical peel solutions are absorbed through the skin, penetrating the epidermis to create and exfoliating effect, and to the dermis, stimulating collagen synthesis. Peels are characterized by the depth of penetration (superficial, medium, and deep). Medium depth peels, such as trichloroacetic acid (TCA), treat dyschromia, keratoses, hyperpigmentation, and fine-to-moderate wrinkles. Deep peels, such as phenol and croton oil, treat deeper, coarser wrinkles. The chosen solution is usually applied until a transparent frost with a pink background is observed, representing penetration to the papillary dermis, as opposed to a solid white frost, which represents the reticular dermis. Eyelids and undermined skin flaps are typically treated with fewer passes and weaker concentrations of the chosen agent. Thicker perioral and forehead skin require more vigorous treatment to attain an opaque frosting color. The most common peel used in our practice during a facelift is the TCA (10%-35%) peel. Benefits include the minimum cost, equipment, and application time. Disadvantages include the operator-dependent depth of penetration and risk of pigmentary changes. If a phenol peel is chosen, there is also a risk for cardiac toxicity, with tachycardia progressing to premature ventricular contractions and then to atrial fibrillation, paroxysmal atrial tachycardia, or ventricular tachycardia.^30,31^ Ideal candidates are light skinned (Fitzpatrick I-III), who have not been treated by medium-to-deep skin resurfacing treatments in the past. Downtime is 2 to 4 weeks.

### Radiofrequency Devices

Radiofrequency energy can be transmitted directly to the dermis and subdermal adipose layer via microneedling applicator, or to the skin and subcutaneous tissues via a bipolar probe. Fractional bipolar microneedling (Morpheus8; InMode, Lake Forest, CA) emits needles at programmable depths depending on the topographic region of the face. The energy leads to dermal coagulation stimulating neocollagenesis and elastogenesis over 4 to 6 weeks. While an anatomical approach to use has been described, surgeons can be guided by light erythema and fine pinpoint bleeding in areas of thick skin such as the perioral region and forehead. A bipolar RF cannula (FaceTite; InMode) provides direct energy through the subcutaneous tissue and skin, coagulating fat near the internal cannula and causing thickening and shrinkage of the collagen FSN, serving to contract the overlying skin. Volumetric studies support shortening of the FSN,^32^ and linear contraction has been reported as high as 31%.^33^ Before the facelift flap is raised, the bipolar cannula can be used on the nasolabial folds and jowls. After the facelift, Morpheus8 microneedling can be used for perioral, periorbital, and forehead zones. The skin flaps themselves can be treated with microneedling, though with modified settings. Standard treatment protocols are provided in Appendices A and B. The benefits of radiofrequency devices are that they can be used on all Fitzpatrick skin types and have minimal downtime and side effects. Most patients are able to return to social engagements in 1 to 3 days. Disadvantages include a high fixed cost initially, and a more modest efficacy of skin resurfacing, with best results achieved with multiple treatments over time (Table).

**Table.** Comparison of Ancillary Procedures During Facelift

Characteristic	Medical grade skin care	Dermabrasion	Ablative lasers	Chemical peels	Radiofrequency bipolar and microneedling
Can be used in all Fitzpatrick skin types	Yes	No	No	No	Yes
Has role as single application during facelift	No	Yes	Yes	Yes	Yes
Continued treatment after facelift possible for further gains	Yes	No	No	No	Yes
Can be used in the context of previous ablative resurfacing	Yes	No	No	Yes	Yes
Can be used in patient who need to return to work within 1-2 weeks	Yes	No	No	No	Yes
Effective for resurfacing fine rhytids	Yes	Yes	Yes	Yes	Yes
Effective for resurfacing moderate/deep rhytids	No	Yes	Yes	No	Yes
No risk of hyperpigmentation	Yes	No	No	No	Yes
No risk of major complications (ie, skin necrosis, systemic complications, etc)	Yes	No	No	No	Yes
No need for antiviral and antibacterial prophylaxis	Yes	No	No	No	Yes

## WHAT HAS CHANGED IN FACELIFT ANCILLARY PROCEDURES: PATIENT DEMAND

Facelifting remains the gold standard for facial rejuvenation. A facelift permits correction of facial skin excess and laxity and repositions the underlying SMAS using one of several techniques depending on the patient's anatomy. However, while facelifting procedures improve the appearance of skin, they do not improve skin quality.

Dermabrasion, various skin peeling agents (phenol, croton oil, and triacetic acid), and lasers (CO2 and ER:YAG) have been used for several decades in the authors’ practice to further improve the appearance (and to some degree the quality) of aged skin during a facelift. These modalities are particularly useful for treating the perioral, periorbital, and forehead areas of the face, which are minimally changed with facelift surgery. These modalities are destructive to the skin, at least to the epidermis and papillary dermis, and have the potential to damage the reticular dermis. They are therefore limited to Fitzpatrick I and II skin types (and occasionally Type III). Hypopigmentation is common, and hyperpigmentation and scarring can likewise occur. Importantly, all these treatment modalities are associated with a skin appearance recovery time of 4 to 6 weeks (and sometimes longer). The benefits of ancillary procedures are compared in Table.

In the past decade, and especially in the past 5 years, there has been increasing demand for facial rejuvenation and body contouring procedures with less healing and downtime. Digital communication with videos, “live” appearances, and photographs of oneself on Facebook, Instagram, TikTok, and dating applications have given rise to a “how do I look at this moment” mentality. This patient population checks social media posts multiple times daily and evaluates everyone else who posts pictures of themselves on social media. Today's patients therefore express a need for a device with minimal healing time in order to return to work and social activity immediately. With respect to facial rejuvenation procedures, patients are happy to accept rejuvenation in multiple sessions with improvements occurring sequentially and over months. After all, multiple sessions have been the longstanding norm for facial fillers, botulism toxin injections, hair color, haircuts, and other frequently performed grooming sessions.

**Figure 1.**
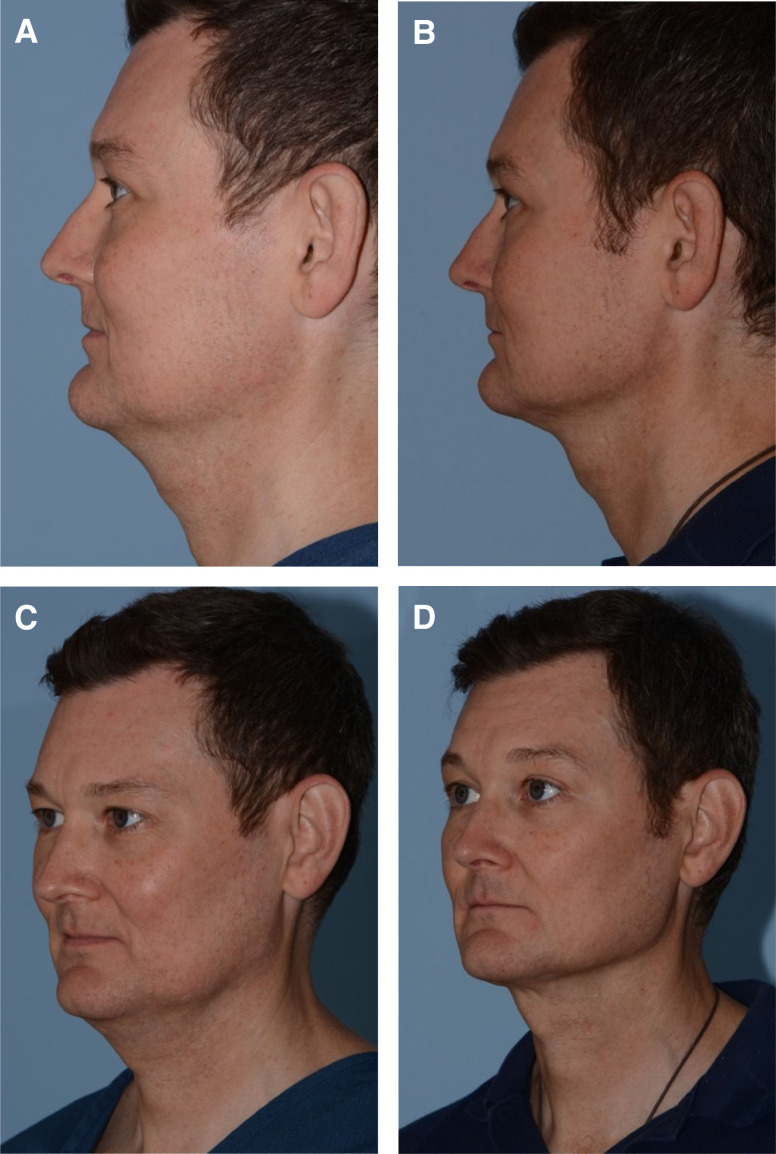
(A, C) Before and (B, D) after FaceTite (InMode; Lake Forest, CA) and Morpheus8 (InMode) neck rejuvenation in a 42-year-old male patient.

**Figure 2.**
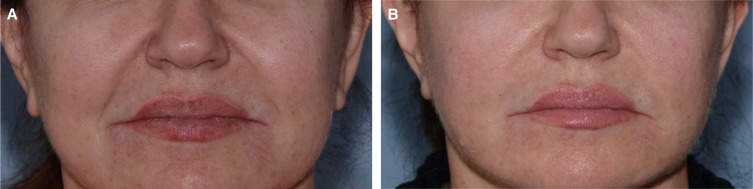
(A) Before and (B) after AcuTite (InMode; Lake Forest, CA) and Morpheus8 (InMode) nasolabial fold rejuvenation in a 60-year-old female patient at the time of a facelift.

**Figure 3.**
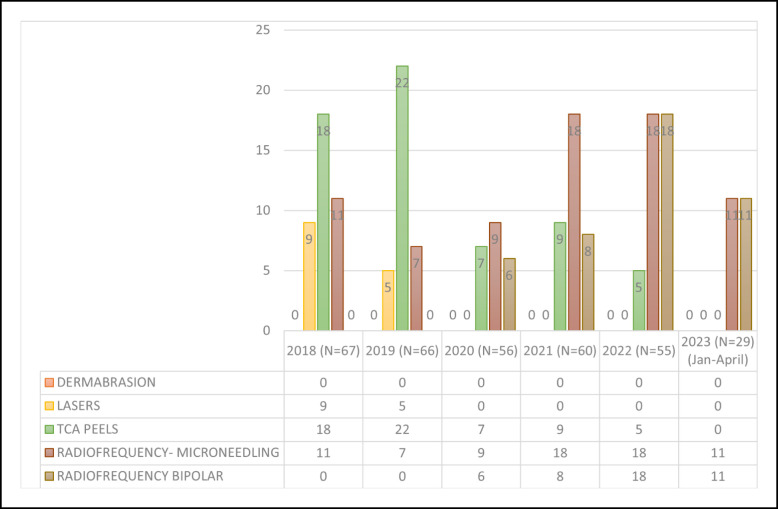
Use of ancillary procedures during facelift from January 2018 to February 2022.

**Figure 4.**
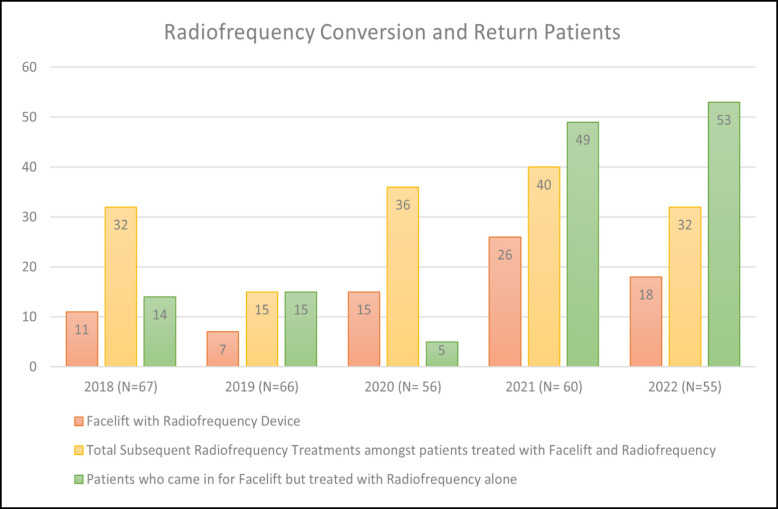
Return and conversion in facelift patients.

Facial rejuvenation using FaceTite and Morpheus8 has increased exponentially in the senior authors’ practice since 2018 (Figures 1-4; Videos 1, 2). These procedures significantly improve facial contour with minimal “recovery,” no sutures, and tolerability under local anesthesia in the office. Furthermore, radiofrequency-assisted microneedling devices such as Morpheus8 improve skin quality and appearance over time. A meaningful radiofrequency microneedling treatment session can be done with the patient able to return to work or social engagements the next day and even the same day. It can safely be performed to the central face during facelifting with normal applicator settings and to undermined flaps under modified settings. The bipolar probe can additionally be used on the nasolabial folds to reduce thickness following skin redraping.

While concomitant FaceTite and Morpheus8 were first limited to “treatment gap” patients (those with laxity not severe enough for a facelift yet not mild enough to treat with noninvasive procedures), over the last 5 years, an increasing proportion of facelift patients are being treated with radiofrequency to further enhance their final outcome.

After becoming acquainted with the technology and seeing the results attainable, it quickly decreased the number of lasers, dermabrasion, and chemical peels performed in the authors’ practice for primary facial rejuvenation. Today, many patients undergo radiofrequency procedures as a stand-alone treatment with minimal downtime recovery period. As the technology advanced, we began to use it for facelift patients which significantly improved our results and allowed us to conclude the following:

1. Radiofrequency microneedling to anterior facial skin (forehead, periorbita, and perioral) that is not undermined during the facelift, significantly improves results, and the majority of these facelift patients returned for subsequent radiofrequency treatments (Figure 4).
2. Microneedling of undermined face and neck skin flaps is safe. It improves skin quality, with approximately 2 days of erythema and without an increase in complications.
3. Treatment of the nasolabial fold before or after flap redraping, first with the bipolar radiofrequency AccuTite probe (InMode), followed by radiofrequency microneedling, reduces the depth of the nasolabial fold definition over and beyond what SMAS and skin redraping can achieve alone.
4. The length of time to first social engagement following facelift surgery with dermabrasion, lasers, and chemical peels is largely a result of the skin resurfacing modality rather than the actual surgery. The recovery time of a facelift with radiofrequency skin treatment is significantly shorter than when ablative skin resurfacing is used (approximately 2 weeks vs 4-6 weeks with concomitant laser or dermabrasion).
5. When describing potential complications of ancillary procedures, today's patients are much less receptive to the possibility of permanent skin changes and are more willing to accept a more modest effect with minimal risk of permanent deformities.

In the authors’ practice, patient demand had made radiofrequency devices the procedure of choice in facial rejuvenation. In the last 5 years, dermabrasion and lasers have been phased out, and the use of chemical peels is waning (Figure 3). Of 67 facelifts in 2018, 11 had Morpheus8 concomitantly with their facelift and 32 more radiofrequency treatments were performed on these patients. Of 66 facelifts in 2019, 7 had Morpheus8 during surgery and 15 additional radiofrequency treatments were performed. Of 56 facelifts in 2020, 9 underwent Morpheus8, 6 FaceTite/AccuTite of the nasolabial folds and 36 subsequent treatments were performed. Of 60 facelifts in 2021, 18 underwent Morpheus8 and 8 FaceTite/AccuTite, and 40 more radiofrequency treatments were performed. Of 55 facelifts in 2022, 18 patients underwent both Morpheus and FaceTite and 32 subsequent treatments were performed.

Dermabrasion had been completely phased out by the data collection period of this study. Lasers were last used on facelift patients in 2018 and 2019 (on 9 and 5 patients, respectively). TCA peels still play a role in the authors’ practice; however, their use is waning (22, 7, 9, and 5 patients from 2019 to 2021, respectively; Figure 3).

## CONCLUSIONS

Ancillary procedures during facelift include dermabrasion, ablative lasers, chemical peels, and radiofrequency devices. All modalities remain safe and effective and should be tailored based on patient anatomy, expectations, and tolerability of side effects and healing time. Over 47 years of using each ancillary modality, the senior author has observed a shift toward a facelift population that demands a shorter recovery with low risk for complications. Radiofrequency devices have addressed shifting demand with a high degree of safety, reliability, and reproducibility. Morpheus8 and FaceTite/AccuTite have become common in the public vernacular and many patients are happy to explain the mild erythema to an acquaintance with “I had Morpheus.” It remains the most common ancillary procedure performed today during facelift in the authors’ practice, and its role in primary nonsurgical facial rejuvenation continues to grow.

## Supplementary Material

ojad063_Supplementary_Data

## References

[1] Li M, Vierkötter A, Schikowski T, et al Epidemiological evidence that indoor air pollution from cooking with solid fuels accelerates skin aging in Chinese women. J Dermatol Sci. 2015;79(2):148–154. doi: 10.1016/j.jdermsci.2015.04.001 26055797

[2] Langton AK, Sherratt MJ, Griffiths CE, Watson RE. A new wrinkle on old skin: the role of elastic fibres in skin ageing. Int J Cosmet Sci. 2010;32(5):330–339. doi: 10.1111/j.1468-2494.2010.00574.x 20572890

[3] Vierkötter A, Schikowski T, Ranft U, et al Airborne particle exposure and extrinsic skin aging. J Invest Dermatol. 2010;130(12):2719–2726. doi: 10.1038/jid.2010.204 20664556

[4] Freiman A, Bird G, Metelitsa AI, et al Cutaneous effects of smoking. J Cutan Med Surg. 2004;8(6):415–423. doi: 10.1007/s10227-005-0020-8 15988548

[5] Velarde MC, Flynn JM, Day NU, Melov S, Campisi J. Mitochondrial oxidative stress caused by Sod2 deficiency promotes cellular senescence and aging phenotypes in the skin. Aging (Albany NY). 2012;4(1):3–12. doi: 10.18632/aging.100423 22278880 PMC3292901

[6] Suter-Widmer J, Elsner P. Age, irritation. In: Agner T, Maibah H, eds. The Irritant Contact Dermatitis Syndrome. CRC Press; 1996:257–265.

[7] Elias PM, Ghadially R. The aged epidermal permeability barrier: basis for functional abnormalities. Clin Geriatr Med. 2002;18(1):103–120, vii. doi: 10.1016/S0749-0690(03)00037-5 11913735

[8] Phillips T, Kanj L. Clinical manisfestations of skin aging. In: Squier C, Hill MW, eds. The Effect of Aging in Oral Mucosa and Skin. CRC Press; 1994:25–40.

[9] Penney KB, Smith CJ, Allen JC. Depigmenting action of hydroquinone depends on disruption of fundamental cell processes. J Invest Dermatol. 1984;82(4):308–310. doi: 10.1111/1523-1747.ep12260588 6200545

[10] Neerken S, Lucassen GW, Bisschop MA, Lenderink E, Nuijs TAM. Characterization of age-related effects in human skin: a comparative study that applies confocal laser scanning microscopy and optical coherence tomography. J Biomed Opt. 2004;9(2):274. doi: 10.1117/1.1645795 15065891

[11] Grove GL. Physiologic changes in older skin. Clin Geriatr Med. 1989;5(1):115–125. doi: 10.1016/S0749-0690(18)30699-2 2645991

[12] Südel KM, Venzke K, Mielke H, et al Novel aspects of intrinsic and extrinsic aging of human skin: beneficial effects of soy extract. Photochem Photobiol. 2005;81(3):581–587. doi: 10.1562/2004-06-16-RA-202.1 15623355

[13] Duncan KO, Leffell DJ. Preoperative assessment of the elderly patient. Dermatol Clin. 1997;15(4):583–593. doi: 10.1016/S0733-8635(05)70468-x 9348459

[14] Krueger N, Luebberding S, Oltmer M, Streker M, Kerscher M. Age-related changes in skin mechanical properties: a quantitative evaluation of 120 female subjects. Skin Res Technol. 2011;17(2):141–148. doi: 10.1111/j.1600-0846.2010.00486.x 21281361

[15] Farage MA, Miller KW, Elsner P, Maibach HI. Functional and physiological characteristics of the aging skin. Aging Clin Exp Res. 2008;20(3):195–200. doi: 10.1007/BF03324769 18594185

[16] Rohrich RJ, Pessa JE. The fat compartments of the face: anatomy and clinical implications for cosmetic surgery. Plast Reconstr Surg. 2007;119(7):2219–2227. doi: 10.1097/01.prs.0000265403.66886.54 17519724

[17] Donofrio LM. Fat distribution: a morphologic study of the aging face. Dermatol Surg. 2000;26(12):1107–1112. doi: 10.1046/j.1524-4725.2000.00270.x 11134986

[18] Wulc AE, Sharma P, Czyz CN. The anatomic basis of midfacial aging. In: Hartstein ME, Wulc AE, Holck DE, eds. Midfacial Rejuvenation. Springer New York; 2012:15–29.

[19] Rohrich RJ, Avashia YJ, Savetsky IL. Prediction of facial aging using the facial fat compartments. Plast Reconstr Surg. 2021;147(1S-2):38S–42S. doi: 10.1097/PRS.0000000000007624 33347073

[20] Dayan E, Rovatti P, Aston S, Chia CT, Rohrich R, Theodorou S. Multimodal radiofrequency application for lower face and neck laxity. Plast Reconstr Surg Glob Open. 2020;8(8):e2862. doi: 10.1097/GOX.0000000000002862 32983756 PMC7489644

[21] Rohrich RJ, Ghavami A, Constantine FC, Unger J, Mojallal A. Lift-and-fill face lift: integrating the fat compartments. Plast Reconstr Surg. 2014;133(6):756e–767e. doi: 10.1097/01.prs.0000436817.96214.7e 24569422

[22] Albert AM, Ricanek K Jr, Patterson E. A review of the literature on the aging adult skull and face: implications for forensic science research and applications. Forensic Sci Int. 2007;172(1):1–9. doi: 10.1016/j.forsciint.2007.03.015 17434276

[23] Smith JE. Dermabrasion. Facial Plast Surg. 2014;30(1):35–39. doi: 10.1055/s-0033-1363759 24488635

[24] Kitzmiller WJ, Visscher M, Page DA, et al The controlled evaluation of dermabrasion versus CO2 laser resurfacing for the treatment of perioral wrinkles. Plast Reconstr Surg. 2000;106(6):1366–1372. doi: 10.1097/00006534-200011000-00024 11083571

[25] Barkana Y, Belkin M. Laser eye injuries. Surv Ophthalmol. 2000;44(6):459–478. doi: 10.1016/S0039-6257(00)00112-0 10906379

[26] Spruance SL. The natural history of recurrent oralfacial herpes simplex virus infection. Semin Dermatol. 1992;11(3):200–206. 1390034

[27] Campbell TM, Goldman MP. Adverse events of fractionated carbon dioxide laser: review of 373 treatments. Dermatol Surg. 2010;36(11):1645–1650. doi: 10.1111/j.1524-4725.2010.01712.x 20961346

[28] Fife DJ, Fitzpatrick RE, Zachary CB. Complications of fractional CO2 laser resurfacing: four cases. Lasers Surg Med. 2009;41(3):179–184. doi: 10.1002/lsm.20753 19291745

[29] Alster TS, Khoury RR. Treatment of laser complications. Facial Plast Surg. 2009;25(5):316–323. doi: 10.1055/s-0029-1243080 20024873

[30] Gross BG. Cardiac arrhythmias during phenol face peeling. Plast Reconstr Surg. 1984;73(4):590–594. doi: 10.1097/00006534-198404000-00012 6709740

[31] Truppman ES, Ellenby JD. Major electrocardiographic changes during chemical face peeling. Plast Reconstr Surg. 1979;63(1):44–48. doi: 10.1097/00006534-197901000-00008 432323

[32] Zelickson BD, Kist D, Bernstein E, et al Histological and ultrastructural evaluation of the effects of a radiofrequency-based nonablative dermal remodeling device: a pilot study. Arch Dermatol. 2004;140(2):204–209. doi: 10.1001/archderm.140.2.204 14967794

[33] Paul M, Mulholland RS. A new approach for adipose tissue treatment and body contouring using radiofrequency-assisted liposuction. Aesthetic Plast Surg. 2009;33(5):687–694. doi: 10.1007/s00266-009-9342-z 19543679 PMC2758217

