# Correction to: Ancillary Procedures to Facelift Surgery: What has Changed?

**DOI:** 10.1093/asjof/ojad087

**Published:** 2023-09-16

This is a correction to: Michael J Stein, Sherell J Aston, Ancillary Procedures to Facelift Surgery: What has Changed?, *Aesthetic Surgery Journal Open Forum*, Volume 5, 2023, ojad063, https://doi.org/10.1093/asjof/ojad063

In the originally published version of this manuscript, the second author’s name was misspelled. The correct spelling of the author’s name is Sherrell J Aston.

This error has been corrected online.

