# Supplementary material for: Ancillary Procedures to Facelift Surgery: What has Changed?
Source: Aesthet Surg J Open Forum. 2023 Aug 16;5:ojad063. doi: 10.1093/asjof/ojad063 (PMC11140481; doi:10.1093/asjof/ojad063)
Supplement: ojad063_Supplementary_Data [file ojad063_Supplementary_Data.zip › 22-0091_Appendix A.docx]

**Appendix A:** Baseline Facial Rejuvenation Settings in Authors Practice

| Anatomical zone | Energy | Depth | Passes | Treatments | Time between treatments |
| --- | --- | --- | --- | --- | --- |
| Forehead | 20-25 | 3mm  2mm  1mm | 1-2  2-3  2-3 | 1-3 | 4 weeks |
| Crows feet | 20-25 | 2mm  1mm | 2-3  2 | 2-3 | 4 weeks |
| Upper eyelids, orbital rim, lower eyelids | 20-25 | 2mm  1mm | 2  2 | 2-3 | 4 weeks |
| Cheeks | 30 | 3mm  2mm  1mm | 3  3  3 | 3-4 | 4 weeks |
| Upper lip, lower lip, chin | 25-30 | 3mm  2mm  1mm | 3  2-1  3-4  3-4 | 3-4 | 4 weeks |
| Fixed perioral resurfacing | 25-30 | 0.5mm | 1-3 | 1-3 | 4 weeks |
| Neck  submental | 25-30 | 3mm  2mm  1mm | 3  3  3 | 3-4 | 4 weeks |
| Nasolabial dold | 25-30 | 4mm  3mm  2mm | 1-2  2-3  2-3 | 2-3 | 4 weeks |
| Jowl | 25 | 3mm  2mm  1mm | 2  2  2 | 1-2 | 4 weeks |
