# Supplementary material for: Ancillary Procedures to Facelift Surgery: What has Changed?
Source: Aesthet Surg J Open Forum. 2023 Aug 16;5:ojad063. doi: 10.1093/asjof/ojad063 (PMC11140481; doi:10.1093/asjof/ojad063)
Supplement: ojad063_Supplementary_Data [file ojad063_Supplementary_Data.zip › 22-0091_Appendix B.docx]

**Appendix B:** Baseline Radiofrequency Settings During Facelift

| Area of the face | Energy | Depth | Number of passes |
| --- | --- | --- | --- |
| Forehead/temples | 30 | 3mm | 3 |
| Periorbita | 20 | 1mm | 3 |
| Perioral | 30 | 2mm | 3 |
| Cheek (undermined flap) | 30 | 2mm  1mm | 1  1 |
| Neck (undermined flap) | 30 | 2mm  1mm | 1  1 |

Note: These settings should be used as a starting point. The authors adjust based on the patient’s age / skin thickness.
